# Supplementary material for: Biomimetic nanocluster photoreceptors for adaptative circular polarization vision
Source: Nat Commun. 2024 Mar 16;15:2397. doi: 10.1038/s41467-024-46646-5 (PMC10944536; doi:10.1038/s41467-024-46646-5)
Supplement: Supplementary file 1 — Supplementary Information [file 41467_2024_46646_MOESM1_ESM.pdf]

Supplementary Information

Biomimetic Nanocluster Photoreceptors for Adaptative Circular Polarization Vision

Wei Wen[1,2,4], Guocai Liu[1,2,4], Xiaofang Wei[1,2,4], Haojie Huang[1,2], Chong Wang[2,3], Danlei Zhu[1,2], Jianzhe Sun[1,2], Huijuan Yan[2,3], Xin Huang[1,2], Wenkang Shi[1,2], Xiaojuan Dai[1,2], Jichen Dong[1,2], Lang Jiang[1,2], Yunlong Guo[1,2], Hanlin Wang[1,2], Yunqi Liu[1,2]

[1]Beijing National Laboratory for Molecular Sciences, CAS Key Laboratory of Organic Solids, Institute of Chemistry, Chinese Academy of Sciences, Beijing 100190, China.
[2]School of Chemical Sciences, University of Chinese Academy of Sciences, Beijing 100049, China.
[3]CAS Key Laboratory of Molecular Nanostructure and Nanotechnology, CAS Research/Education Center for Excellence in Molecular Sciences, Beijing National Laboratory for Molecular Science, Institute of Chemistry, Chinese Academy of Sciences, Beijing 100190, China.
[4]These authors contributed equally: Wei Wen, Guocai Liu, Xiaofang Wei.
Corresponding authors. E-mails: wanghanlin@iccas.ac.cn; liuyq@iccas.ac.cn

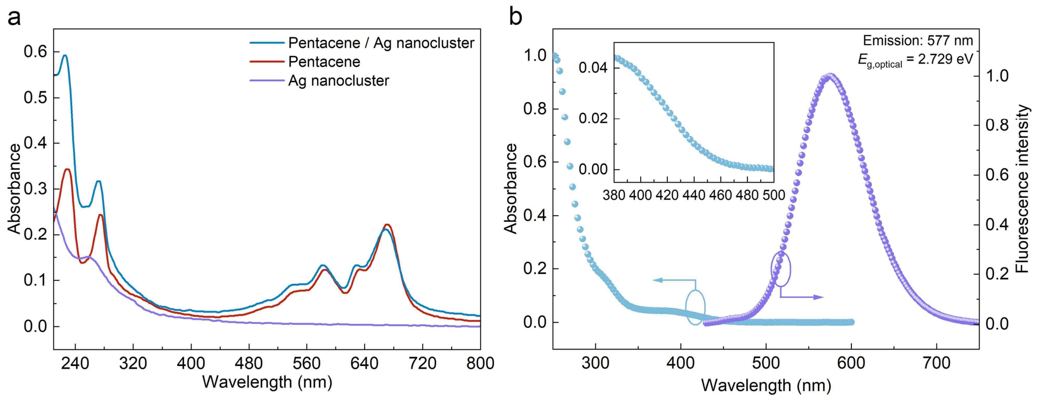

Supplementary Fig. 1 | Spectral characterization of Ag nanoclusters and pentacene. (a) UV-vis spectra of Ag nanoclusters and pentacene. Ag nanoclusters have strong absorption of UV light while pentacene has a wide range absorption from UV to red light. **(b)** UV-vis spectra and fluorescence spectra of Ag nanoclusters. Its optical gap is calculated as 2.729 eV and peak emission wavelength is located at 577 nm. Inset, the enlarged image of UV-vis spectra of nanoclusters.

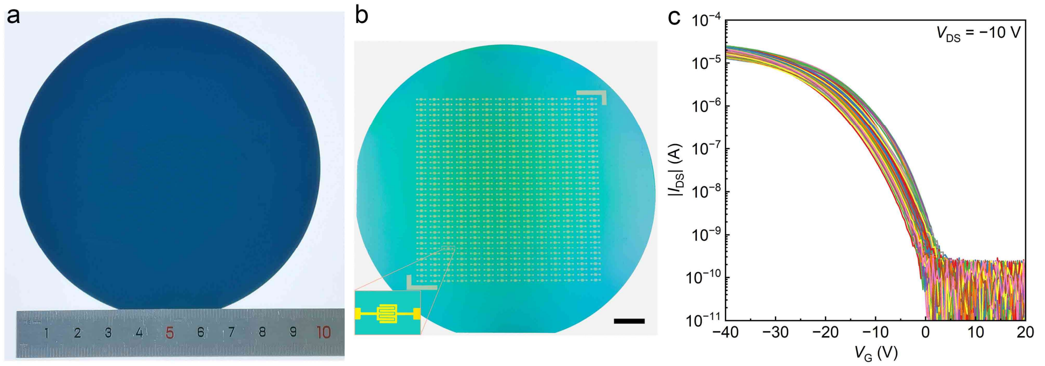

Supplementary Fig. 2 | Images and electrical characteristics of an ACP array. (a) Optical microscope image of 4-inch wafer-scale Ag nanocluster film. **(b)** Image of a 4-inch wafer-scale ACP array. Scale bar: 1 cm. Channel width and length of ACP are 4,500 μm and 20 μm, respectively. **(c)** Transfer characteristics of arbitrarily chosen 200 devices on the wafer were measured under dark condition at V_{DS} = −10 V and they display a high degree of uniformity.

a

b

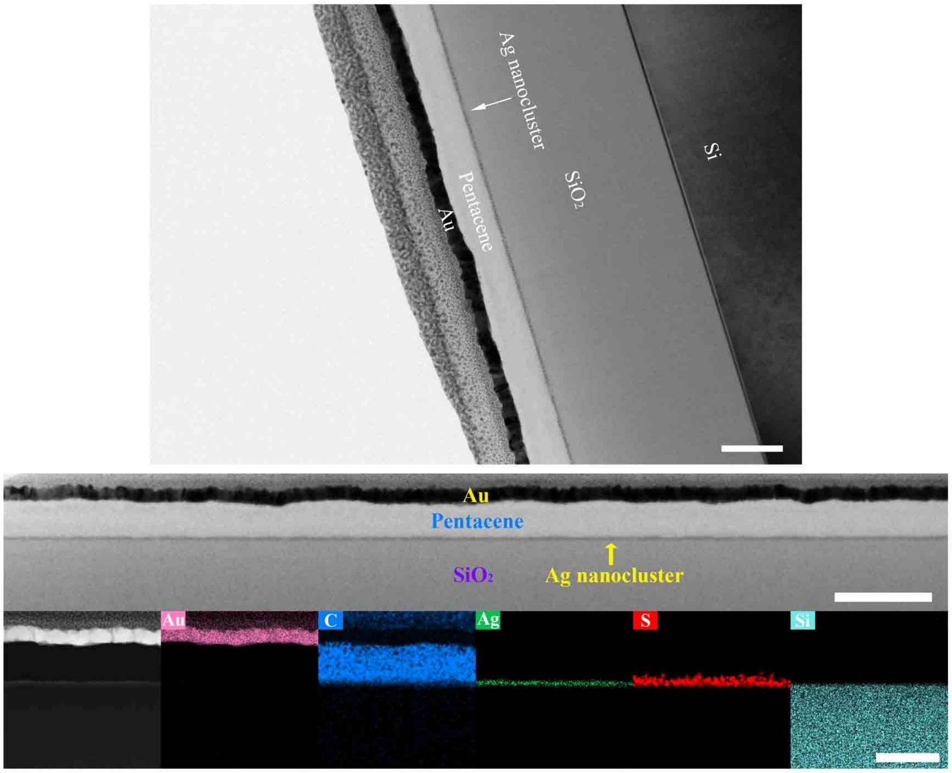

Supplementary Fig. 3 | Cross-sectional TEM image of an ACP. (a) low-magnification cross-sectional TEM image of an ACP. Scale bar: 100 nm. **(b)** Cross-sectional SEM image (Scale bar: 200 nm) and corresponding EDS mapping (Scale bar: 100 nm) of an ACP. Ag nanocluster-pentacene heterojunction is clearly identified by its interface.

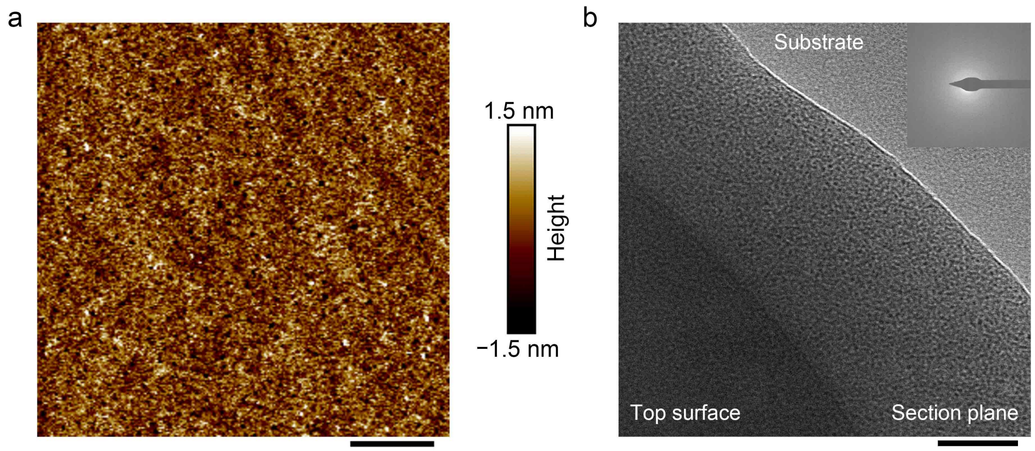

Supplementary Fig. 4 | AFM and Cryo-transmission electron microscope images of Ag nanocluster film. (a) AFM height image of Ag nanocluster film with a root-mean-square roughness of 0.45 nm. Scale bar: 1 μm. **(b)** Cryo-transmission electron microscope image of the edge of Ag nanocluster film. Scale bar: 40 nm. Ag nanoclusters pack arbitrarily and tightly.

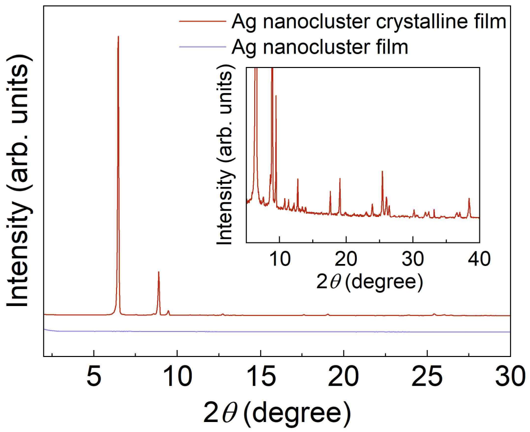

Supplementary Fig. 5 | XRD patterns of the Ag nanocluster film. The blue line: Ag nanocluster film is prepared by high-speed spin-coating method mentioned in manuscript to suppress the crystallization process. The orange line: Ag nanocluster film is formed by dipping volatile Ag nanocluster solution on substrate. Inset, the enlarged image of XRD pattern of Ag nanocluster crystalline film.

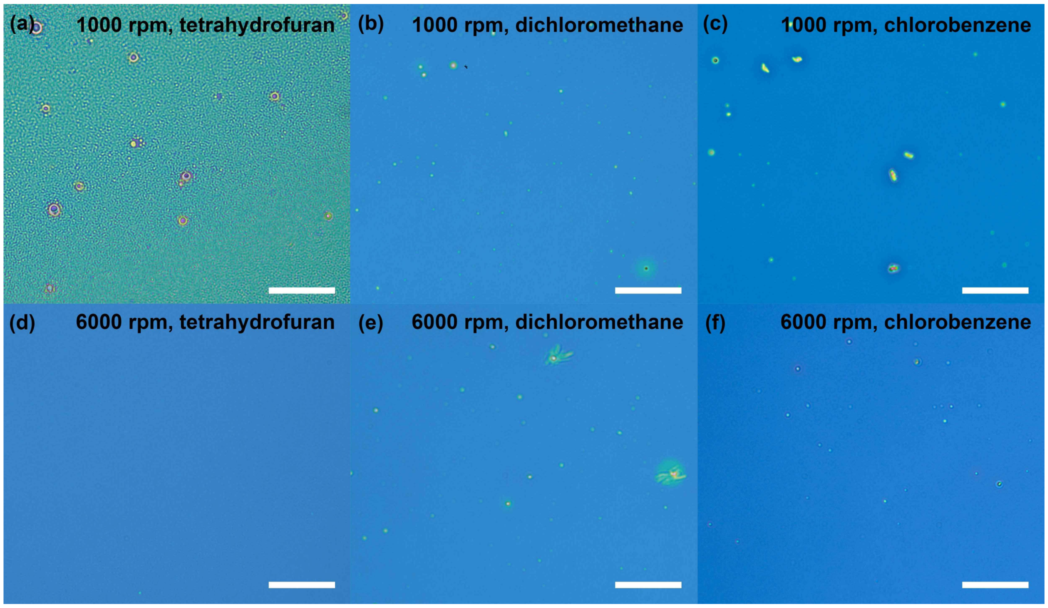

Supplementary Fig. 6 | Optical microscope images of nanocluster films obtained from various spin-coating recipes. (a) Tetrahydrofuran solvent with 1000 rpm spin-speed**. (b)** Dichloromethane solvent with 1000 rpm spin-speed**. (c)** Chlorobenzene solvent with 1000 rpm spin-speed**. (d)** Tetrahydrofuran solvent with 6000 rpm spin-speed**. (e)** Dichloromethane solvent with 6000 rpm spin-speed**. (f)** Chlorobenzene solvent with 6000 rpm spin-speed**.** High spin-speed facilitates the quality of Ag nanocluster film. Tetrahydrofuran is a proper solvent to form a uniform nanocluster film. Scale bar: 20 μm.

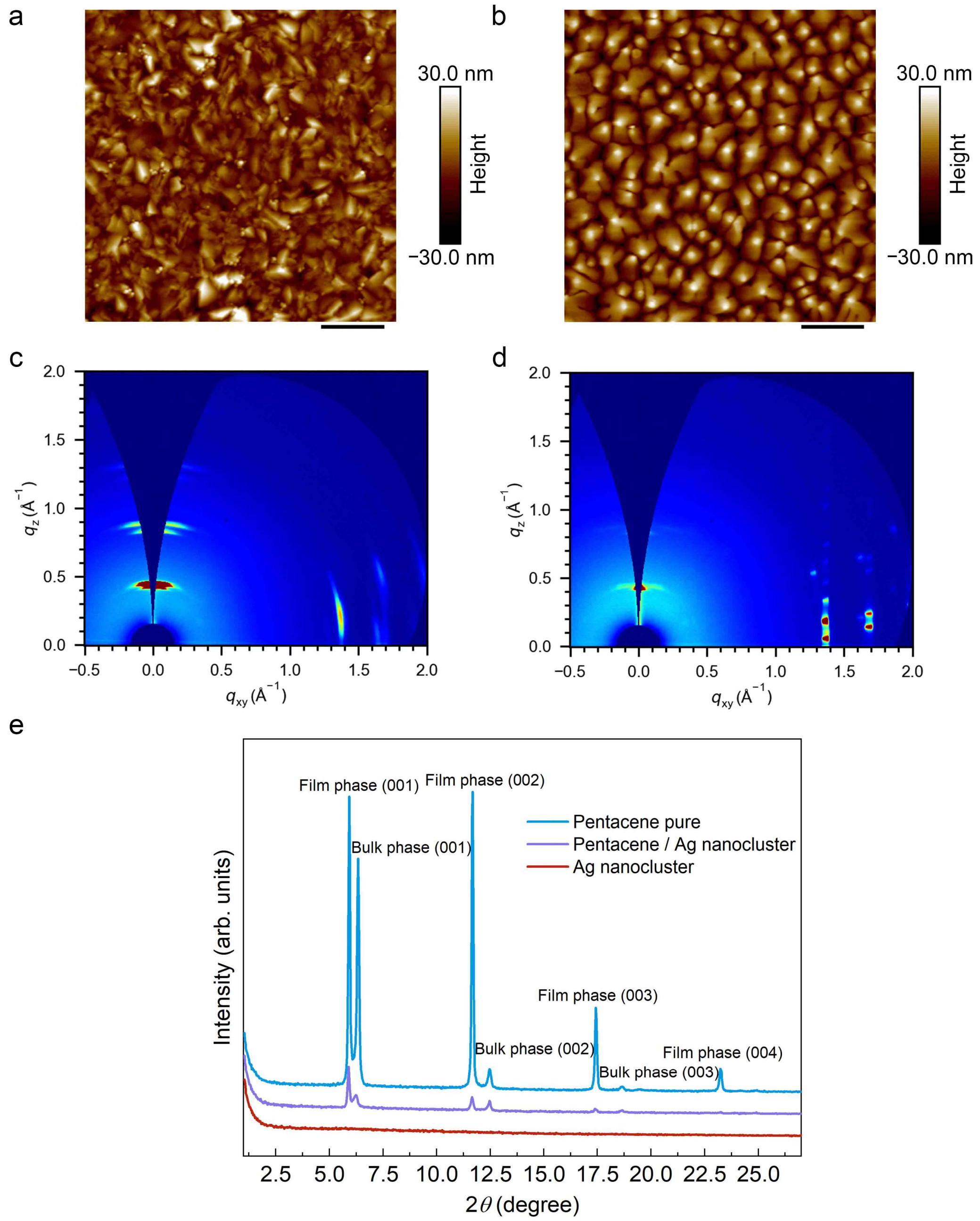

Supplementary Fig. 7 | Characteristics of pentacene film. (a) AFM image of pentacene deposited on Ag nanocluster film with a root-mean-square roughness of 6.82 nm. Scale bar: 1 μm. **(b)** AFM image of pentacene deposited on SiO_2 with a root-mean-square roughness of 9.17 nm. Scale bar: 1 μm. Pentacene deposited on Ag nanoclusters displays lower roughness and reduced grain sizes compared to its analogue deposited directly on SiO_2. **(c)** GIWAXS image of pentacene deposited on Ag nanocluster film. **(d)** GIWAXS image of pentacene deposited on SiO_2. Lattice spacing and π-π stacking distances of pentacene films nearly remain consistent in two conditions. **(e)** XRD of the Ag nanocluster film, pentacene film deposited on Ag nanoclusters and pentacene film deposited on SiO_2. Ag nanocluster film shows no diffraction peaks, while crystalline

degree of pentacene/Ag nanoclusters is notably lower than neat pentacene.

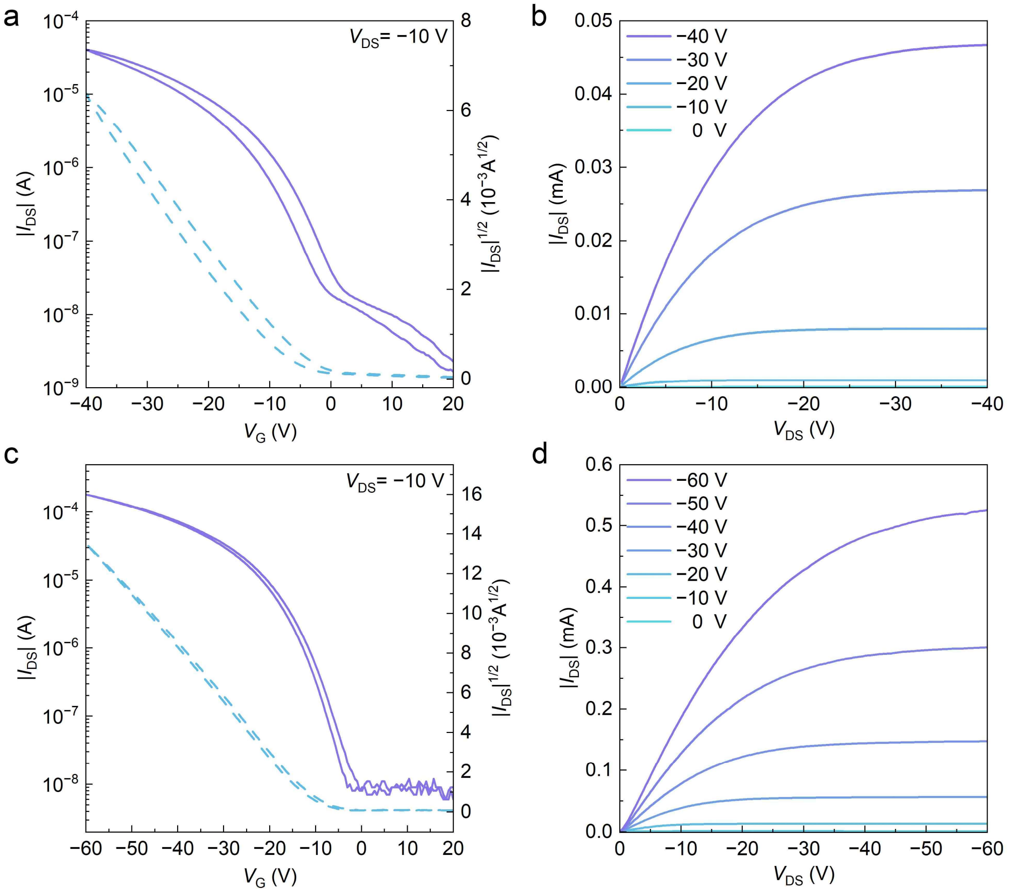

Supplementary Fig. 8 | Electric characteristics of devices. (a, b) Transfer and output characteristic curves of an ACP device. **(c, d)** Transfer and output characteristic curves of a neat pentacene OFET.

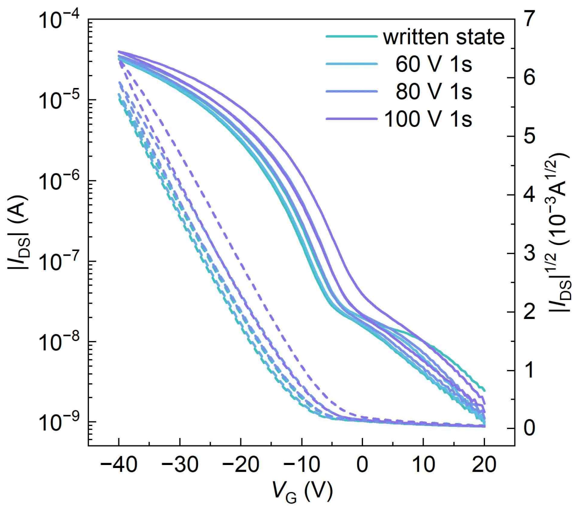

Supplementary Fig. 9 | Transfer characteristic curve of an ACP device after positive V_G impulses under darkness. Threshold voltage (V_{th}) of ACP shifts to the positive direction.

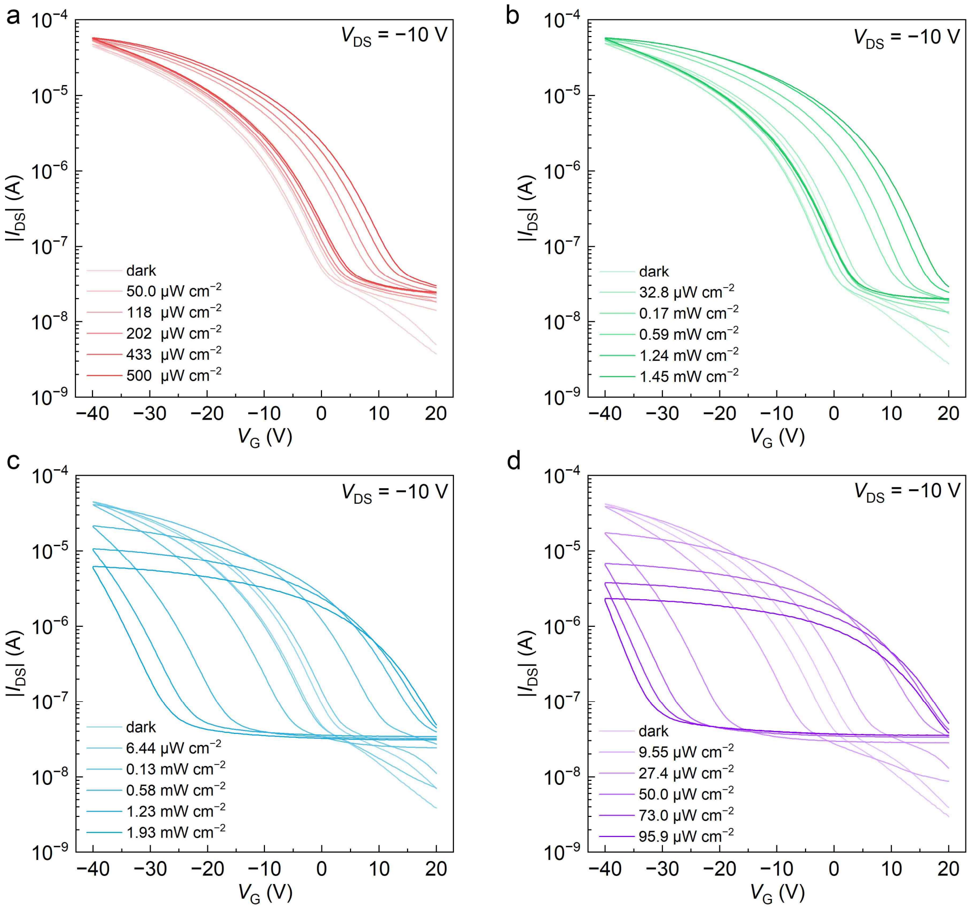

Supplementary Fig. 10 | Hysteresis loop of ACP under illumination with different wavelengths and light densities. (a) Red light (625 nm). **(b)** Green light (525 nm). **(c)** Blue light (460 nm). **(d)** UV light (365 nm).

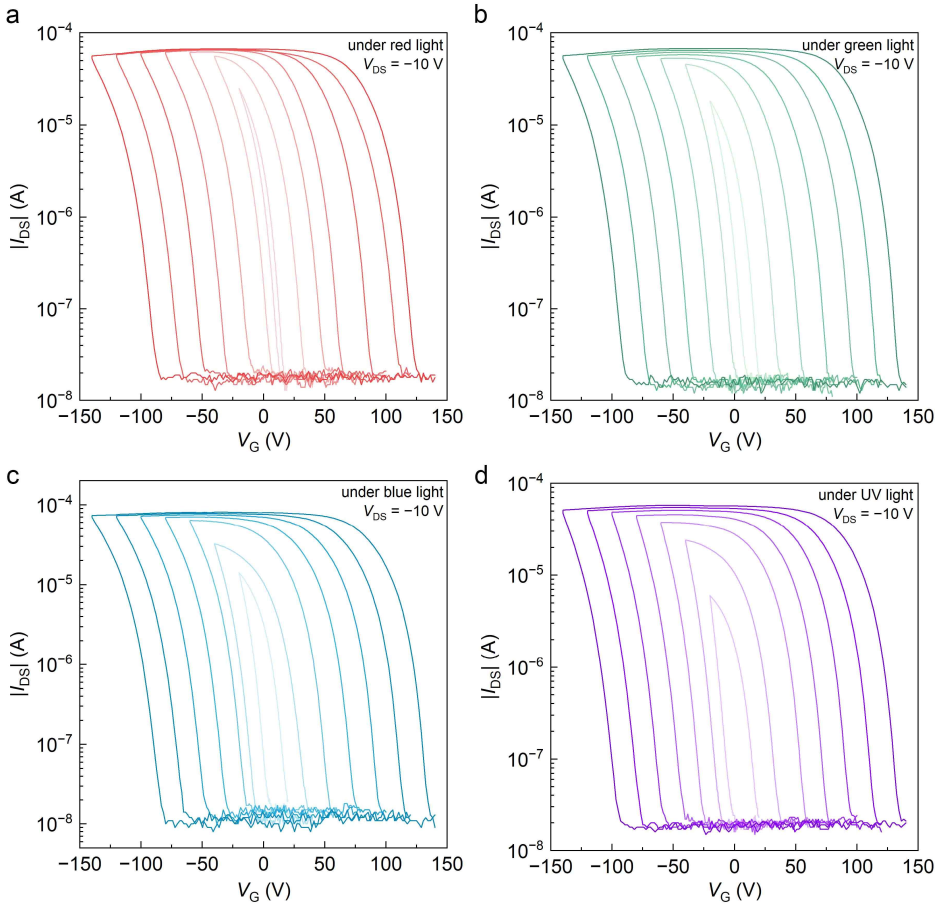

Supplementary Fig. 11 | Light memory with various scanning ranges of V_G. (a) Red light (625 nm, 2 mW cm^{-2}). **(b)** Green light (525 nm, 2 mW cm^{-2}). **(c)** Blue light (460 nm, 2 mW cm^{-2}). **(d)** UV light (365 nm, 100 μW cm^{-2}). Irrespective of light wavelengths, memory window can reach up to 214 V with the increase in scanning range and light intensities.

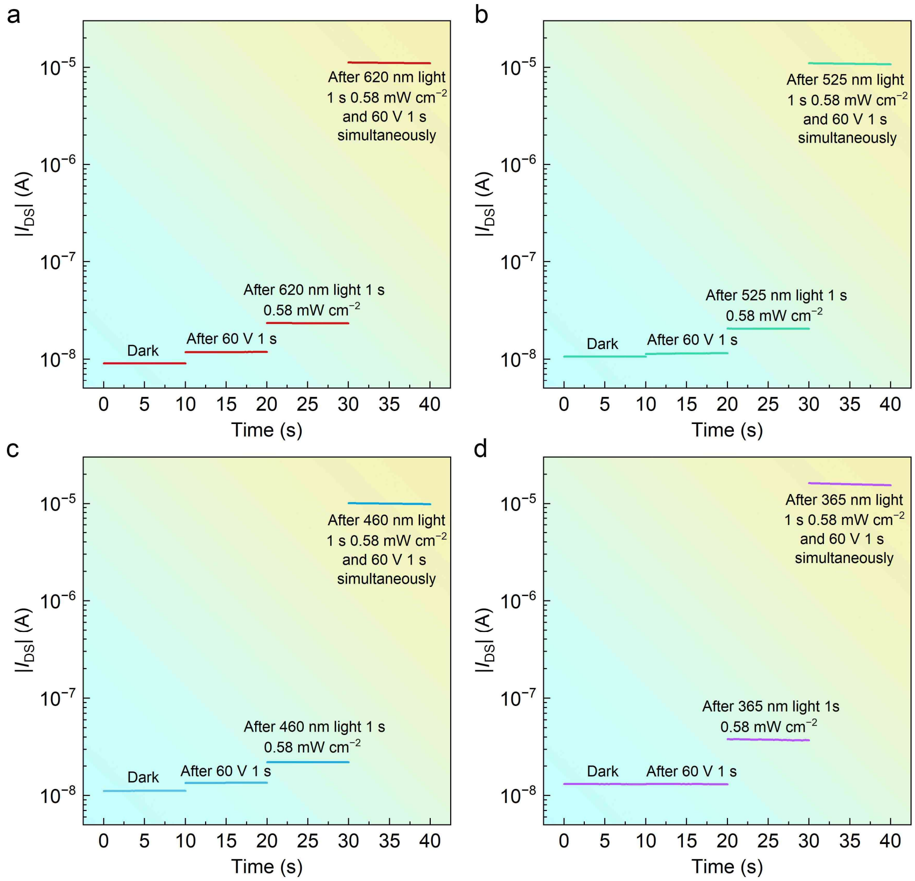

Supplementary Fig. 12 | Current levels in an ACP after operations. The ACP can be modulated by electric impulses, light impulses and conjoint modulation of electric and light stimuli. V_G pulses are applied as 60 V for 1 s, while light pulses are applied with various wavelengths. **(a)** Red 620 nm. **(b)** Green 525 nm. **(c)** Blue 460 nm. **(d)** UV 365 nm. Duration of light impulses is 1 s for all figures.

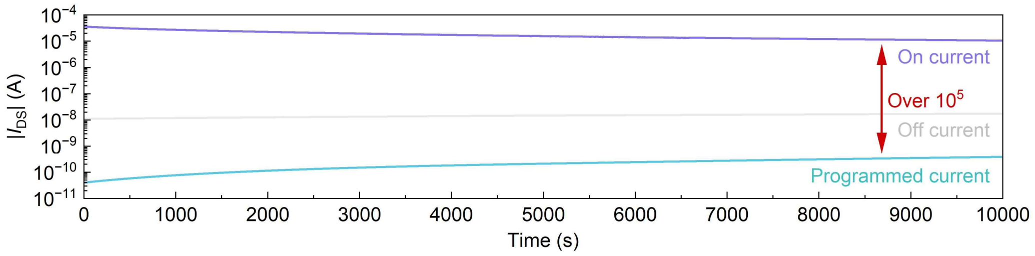

Supplementary Fig. 13 | I_{DS} retention within a timescale of 10,000 s. On-state current is measured after an applied pulse (V_G = 60 V and 460 nm, 2 mW cm^{-2} simultaneously applied for 1s). Off-state current is measured in darkness without application of V_G biasing. Programmed current is measured after an applied pulse (V_G = −60 V, duration of 1 s).

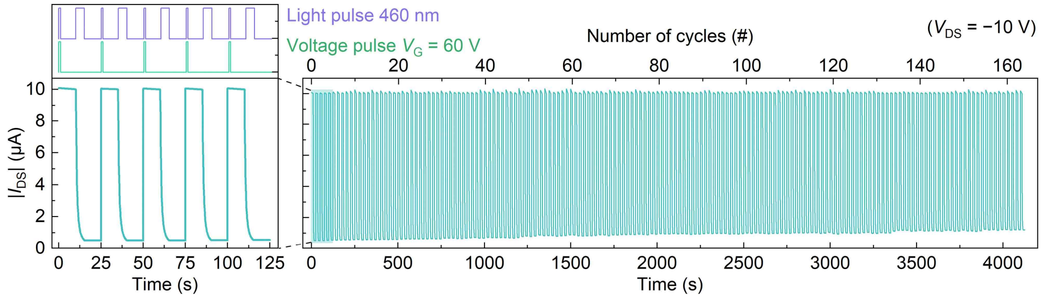

Supplementary Fig. 14 | Cycling endurance test for 165 cycles. Each cycle contains photoresponse, signal retention and photoadaptation processes. Photoreponse is achieved by concurrent impulses of V_G (60 V, 1 s) and 460 nm LED (2 mW cm^{-2}, 1 s). Signal retention retains for 10s with V_G = 0 V under darkness. 460 nm light (2 mW cm^{-2}) stimulated for 5 s alone produces photoadaptation. V_{DS} is kept constant at −10 V.

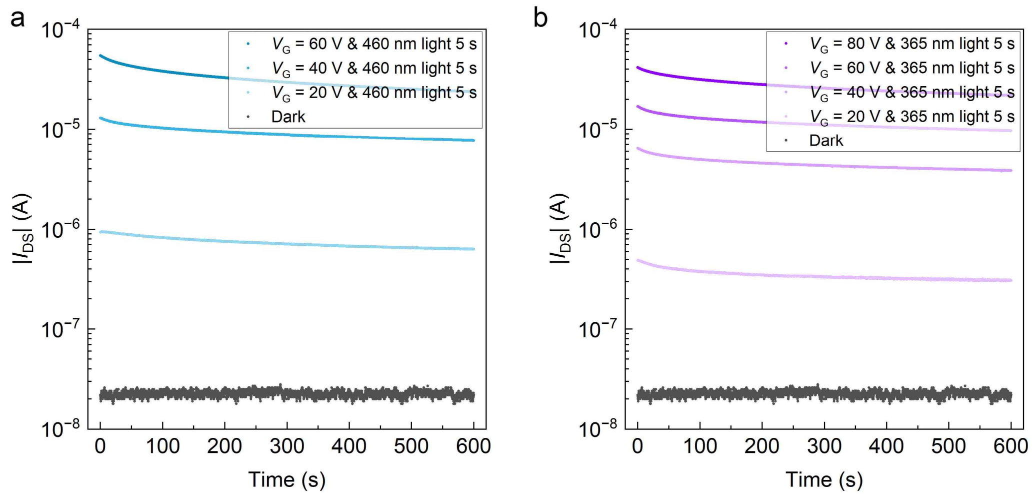

Supplementary Fig. 15 | Multi-level light-stimulated storage in an ACP. Conjoint modulation of V_G pulse and **(a)** 460 nm (blue) light, **(b)** 365 nm (UV) light stimuli enable multi-level retention signals in an ACP.

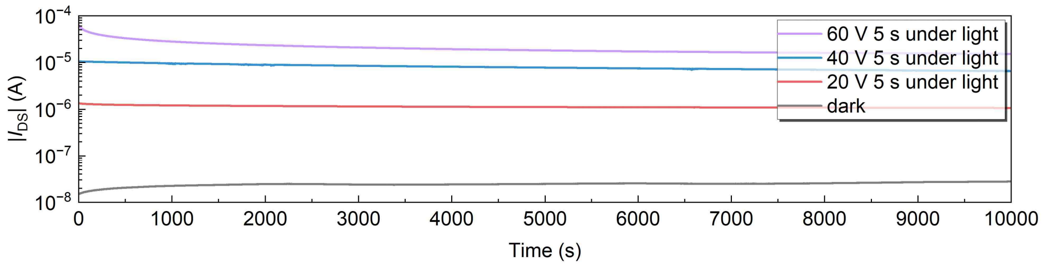

Supplementary Fig. 16 | Multi-level retention within a timescale of 10,000 s. Conjoint modulation of V_G pulse and 460 nm (blue) light stimuli enable multi-level retention signals in an ACP. Note that this device has been stored in a N_2-filled glovebox for one year prior to electrical characterization.

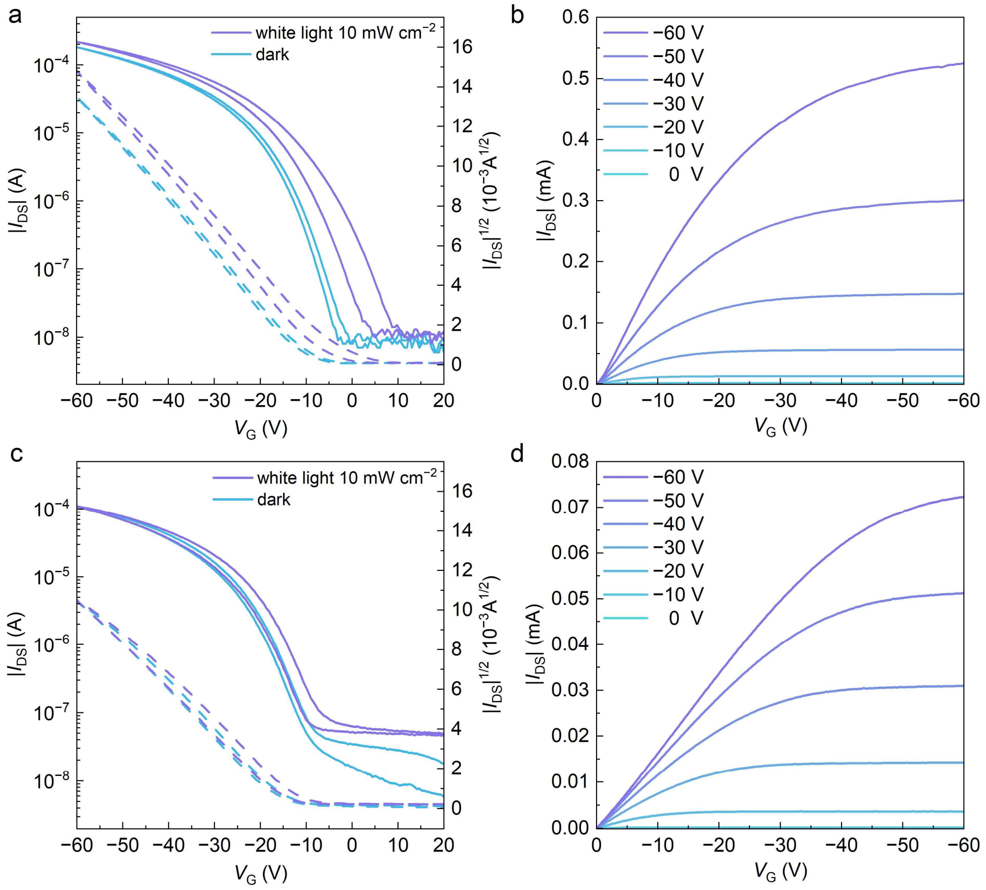

Supplementary Fig. 17 | Transfer and output characteristics of device A and device B for control experiments. (a, b) Device A: pentacene/SiO_2 device without the use of Ag nanocluster layer. **(c, d)** Device B: an ACP with a supplementary layer of 7 nm tetratetracontane sandwiched between pentacene and Ag nanoclusters, which is highly similar to a conventional floating-gate transistor. Tetratetracontane here functions as the tunneling layer.

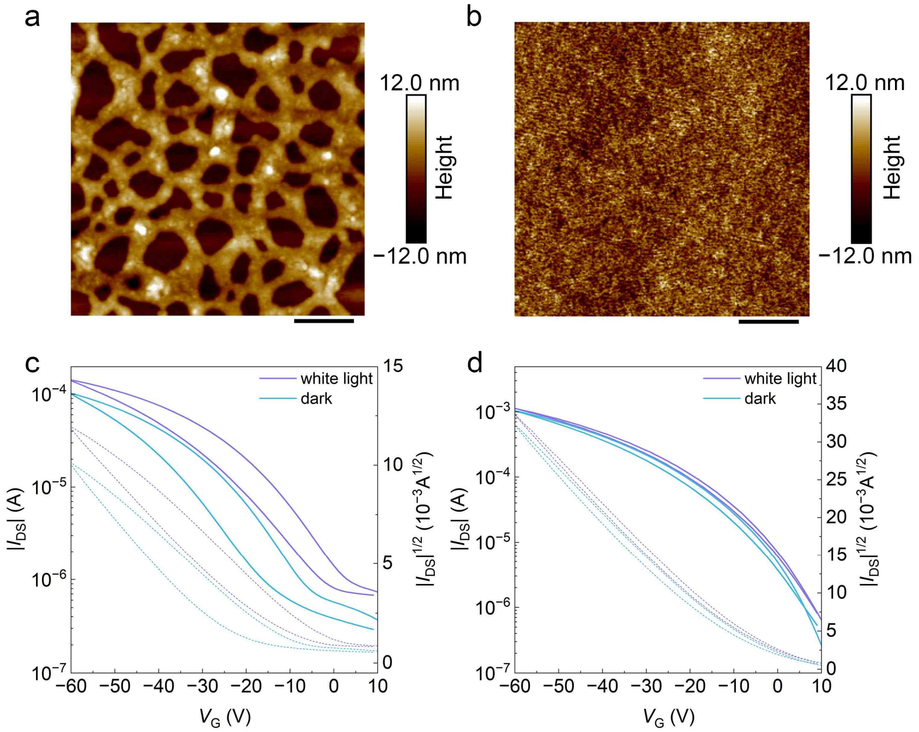

Supplementary Fig. 18 | Electrical properties and morphology of quantum-dot-interfaced pentacene devices. (a) AFM of PbS dot film with a root-mean-square roughness (R_{RMS}) of 4.34 nm. Scale bar: 400 nm. (b) AFM of carbon dot film with a R_{RMS} of 0.248 nm. Scale bar: 1 μm. (c) Bidirectional transfer characteristics of PbS dots-based photoreceptors. (d) Bidirectional transfer characteristics of carbon dots-based photoreceptors. These two types of devices show inferior photoperception capabilities.

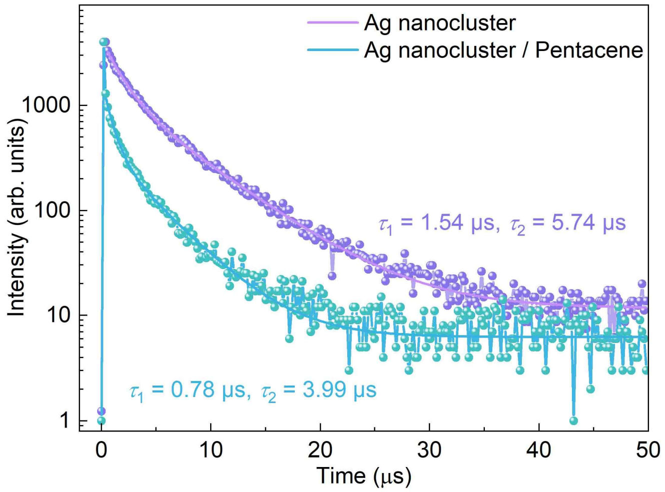

Supplementary Fig. 19 | Time-resolved photoluminescence (TRPL) spectra of Ag nanoclusters and pentacene/Ag nanoclusters. The experimental curves can be fitted by exponential decay equation as below:

$$y = y_0 + A_1 e^{-x/\tau_1} + A_2 e^{-x/\tau_2} \tag{1}$$

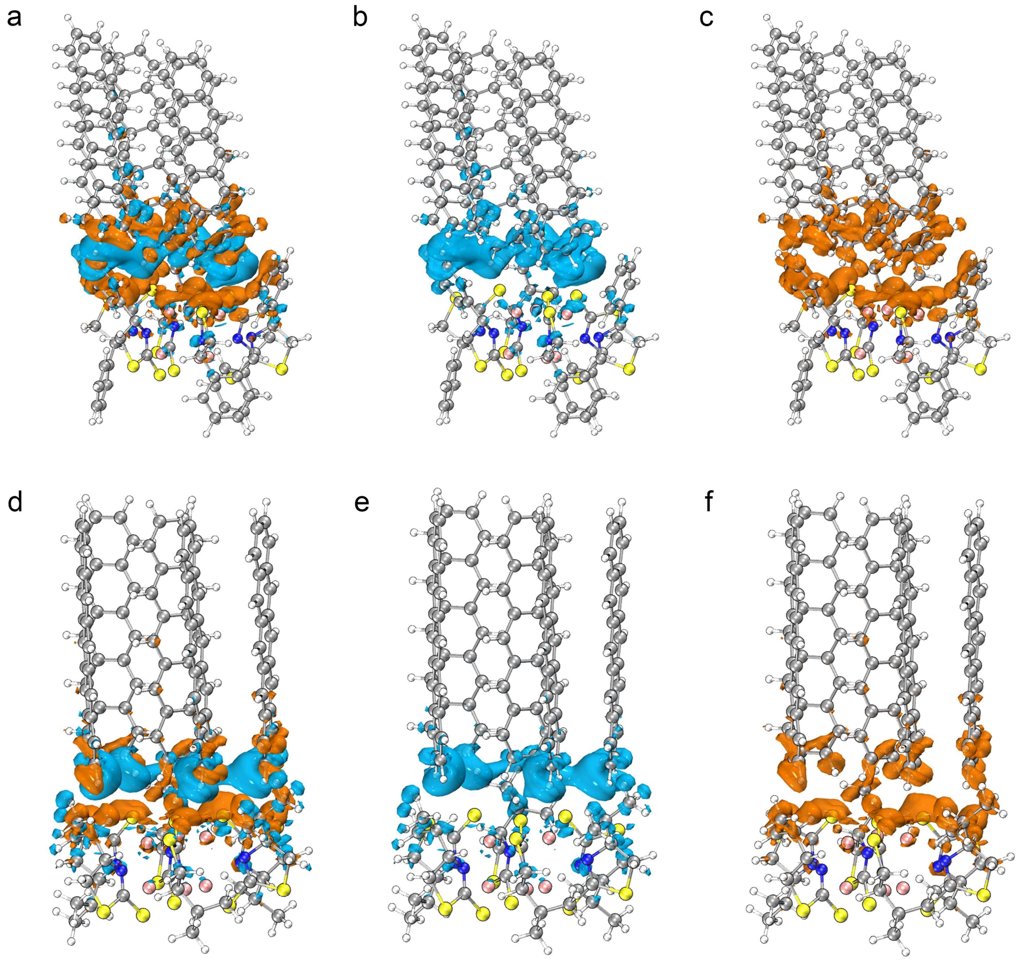

Supplementary Fig. 20 | Charge density difference of the pentacene/Ag nanocluster heterostructure. (a-c) Ag nanoclusters protected by phenyl ligands. **(d-f)** Ag nanoclusters protected by alkyl ligands. The blue and orange colors indicate the negative and positive values, respectively. The isosurface value is 0.002 e/$Å^{-3}$.

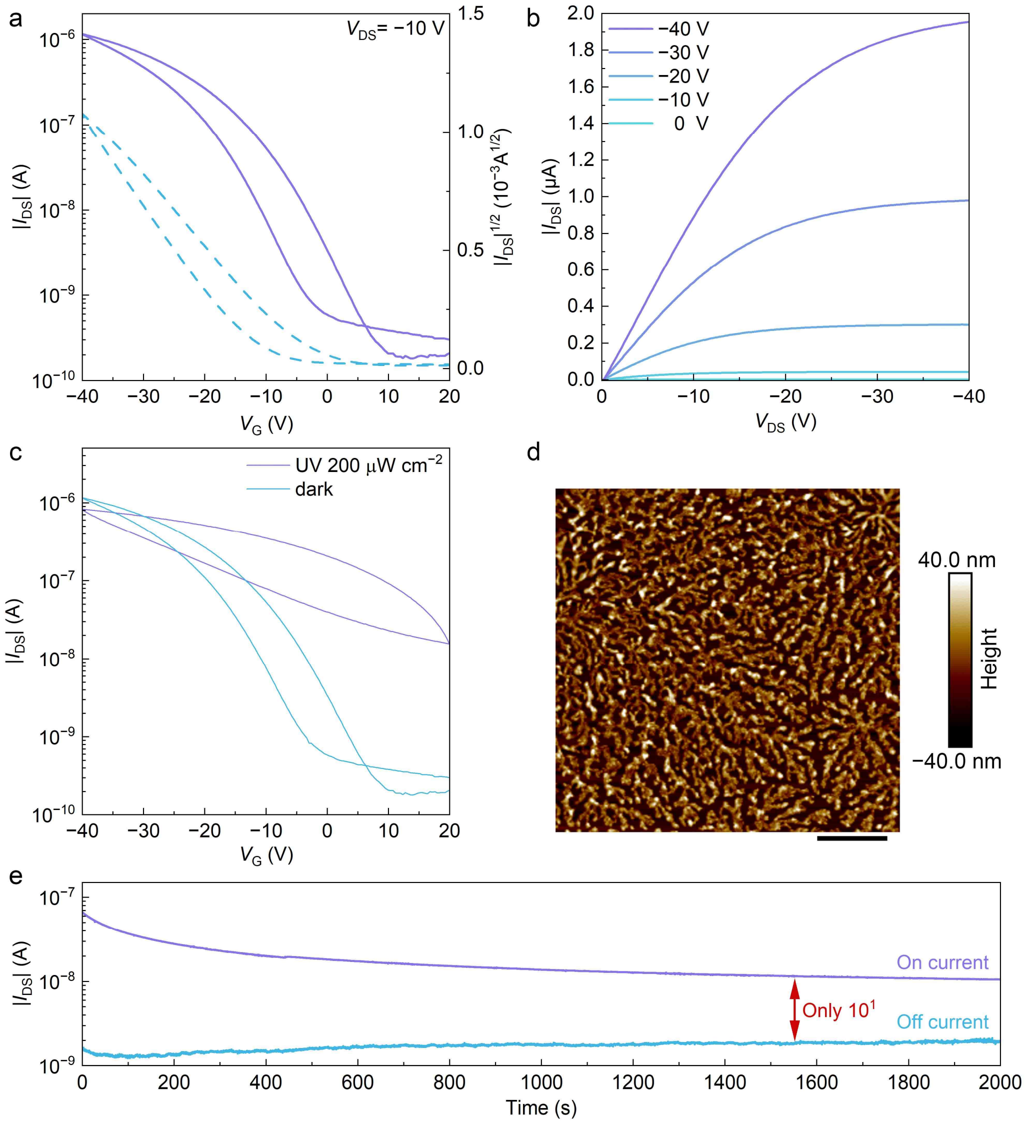

Supplementary Fig. 21 | Electrical properties and morphology of alkyl ligand protected Ag nanocluster devices. (a) Bidirectional transfer characteristics. **(b)** Output characteristics. **(c)** Bidirectional transfer characteristics curve under dark and light conditions. **(d)** AFM of alkyl ligand protected nanocluster film. Scale bar: 2 μm. **(e)** I_{DS} retention within a timescale of 2,000 s.

The aim of presenting two types of nanoclusters is to clarify the structure-performance correlation in ligands. To our knowledge, few research articles have probed the charge-transfer characteristics at the nanocluster/organic semiconductor interface, let alone the role ligands played in signal transduction. It is widely acknowledged that charge transport along co-facially stacked π planes present a critical pathway in organic semiconductors. Hence, it can be inferred that aromatic ligands other than alkyl ligands have higher structure affinity to conjugated backbones in

organic semiconductors, enabling more efficient charge transport at the ligand/organic semiconductor interface and potentially lower injection barrier. Meanwhile, the less ‘conductive’ alkyl chain would lead to electron tunneling rather than hopping that is readily available in aromatic ligands. Furthermore, we have proved our speculation by experiments and theoretical calculation. The alkyl ligand protected nanoclusters have notably inferior performance in memory capacity and retention time, being due to the following reasons:

First, aromatic ligands function as a percolation pathway to transport charges, instead alkyl ligands break the coherence of π systems. Second, we find that two kinds of nanoclusters have slight difference in energy levels, which in turn governs the interfacial injection barrier or efficiency. The highest occupied molecule orbitals (HOMO) of nanoclusters are estimated from ultraviolet photoelectron spectroscopy (UPS) and the lowest un-occupied molecular orbitals (LUMO) are calculated from optical band gap, as shown in Supplementary Fig. 22 and Supplementary Table 1. Third, ligand-induced morphology evolution of nanoclusters. We find that the root-mean-square roughness of the alkyl ligand protected nanocluster film is 13.2 nm, being much higher than the aromatic one, which is responsible for non-uniformity, interfacial defect formation with high density and deteriorated performance.

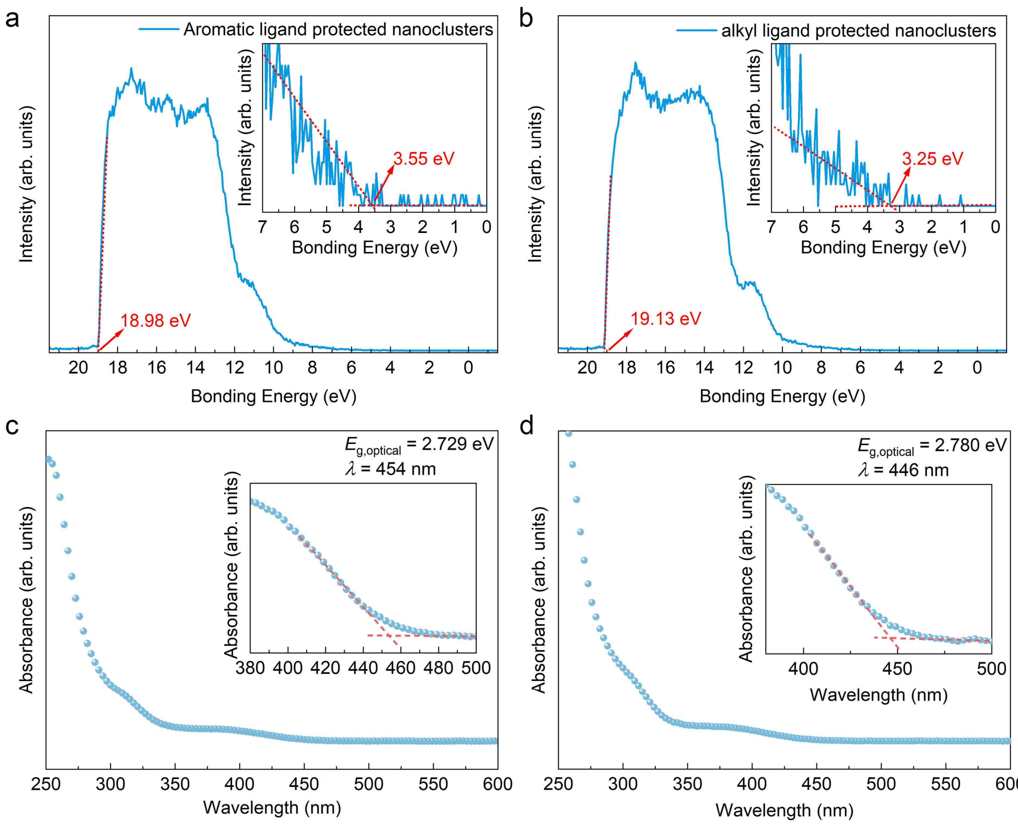

Supplementary Fig. 22 | UPS and UV-vis spectra of nanocluster films. (a) UPS spectra of aromatic ligand protected nanoclusters. Inset, the enlarged image of UPS spectra. **(b)** UPS spectra of alkyl ligand protected nanocluster films. Inset, the enlarged image of UPS spectra. **(c)** UV-vis spectra of aromatic ligand protected nanoclusters. Inset, the enlarged image of UV-vis spectra. **(d)** UV-vis spectra of alkyl ligand protected nanocluster films. Inset, the enlarged image of UV-vis spectra.

Supplementary Table 1 | Summary of energy levels of Ag nanoclusters.

	Aromatic ligand protected	Alkyl ligand protected
HOMO (eV)	−5.79	−5.34
LUMO (eV)	−3.06	−2.56
Eg (eV)	2.73	2.78

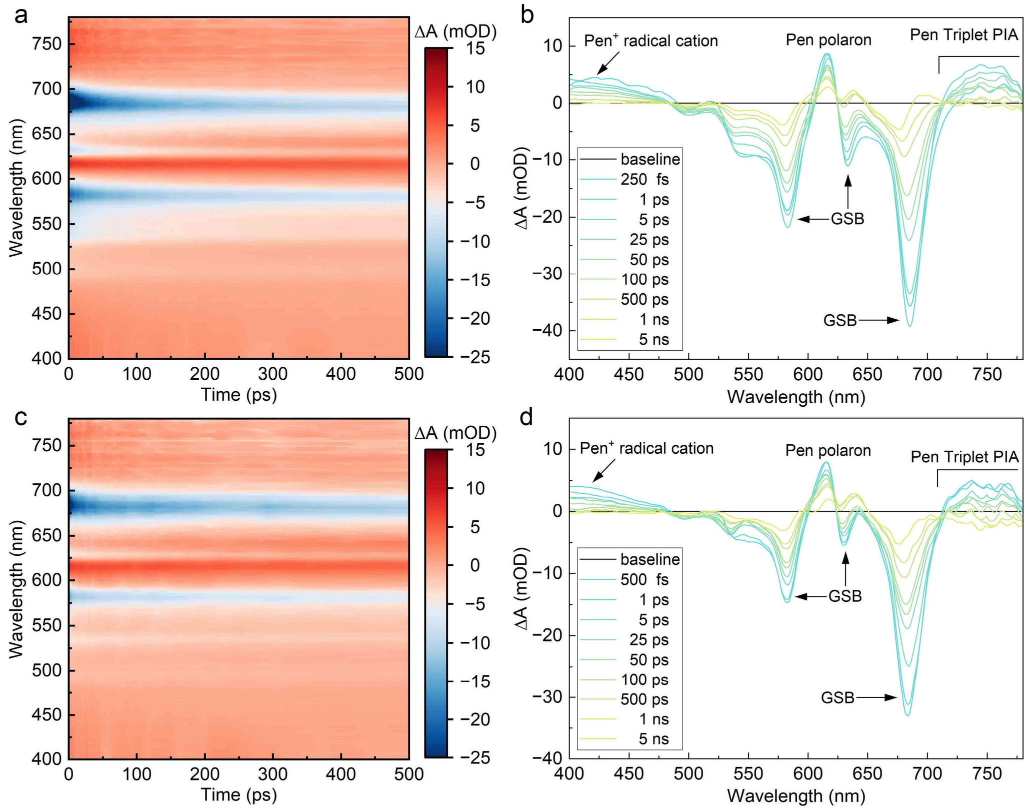

Supplementary Fig. 23 | Femtosecond transient absorption. (a) and **(c)** are pentacene and pentacene/Ag nanocluster films excited at 580 nm, respectively. **(b)** and **(d)** are extracted from **(a)** and **(c)**, respectively. Pen is the abbreviation of Pentacene. GSB: ground-state bleaching; PIA: photo-induced absorption.

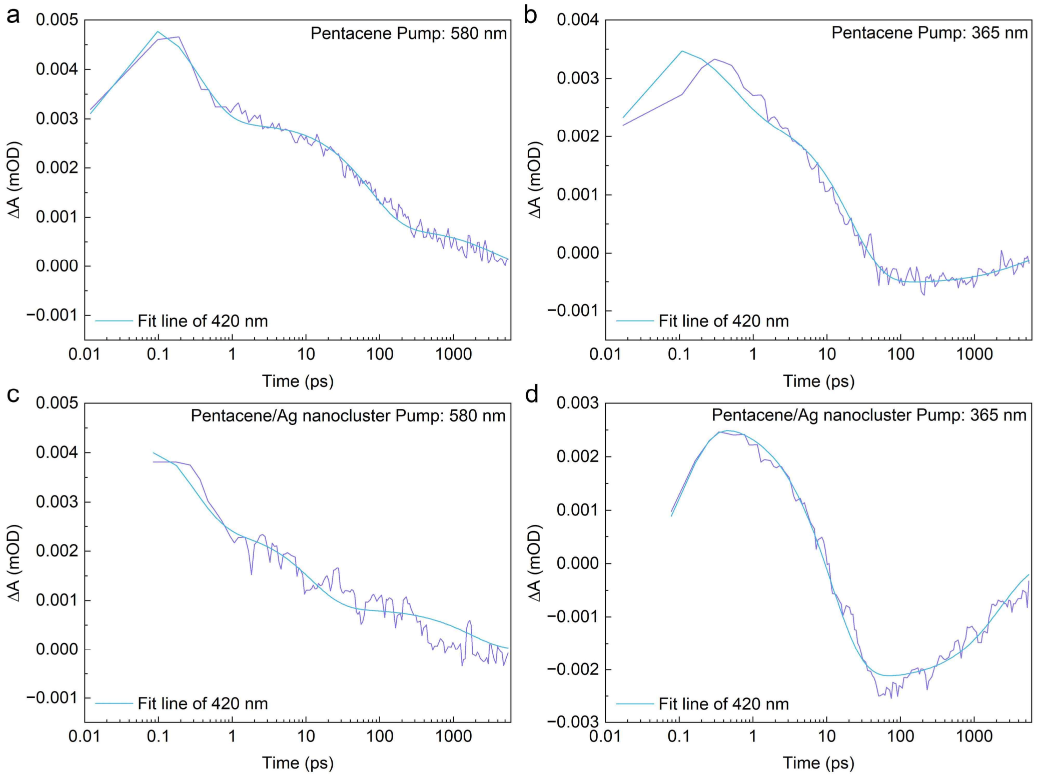

Supplementary Fig. 24 | Time absorption profile analyzed from global analysis at 420 nm wavelength. (a) pentacene film is pumped by 580 nm light. **(b)** pentacene film is pumped by 365 nm light. **(c)** pentacene/Ag nanocluster film is pumped by 580 nm light. **(d)** pentacene/Ag nanocluster film is pumped by 365 nm light. Fitted kinetic curves are in good agreement with the raw data, demonstrating the suitability of global analysis.

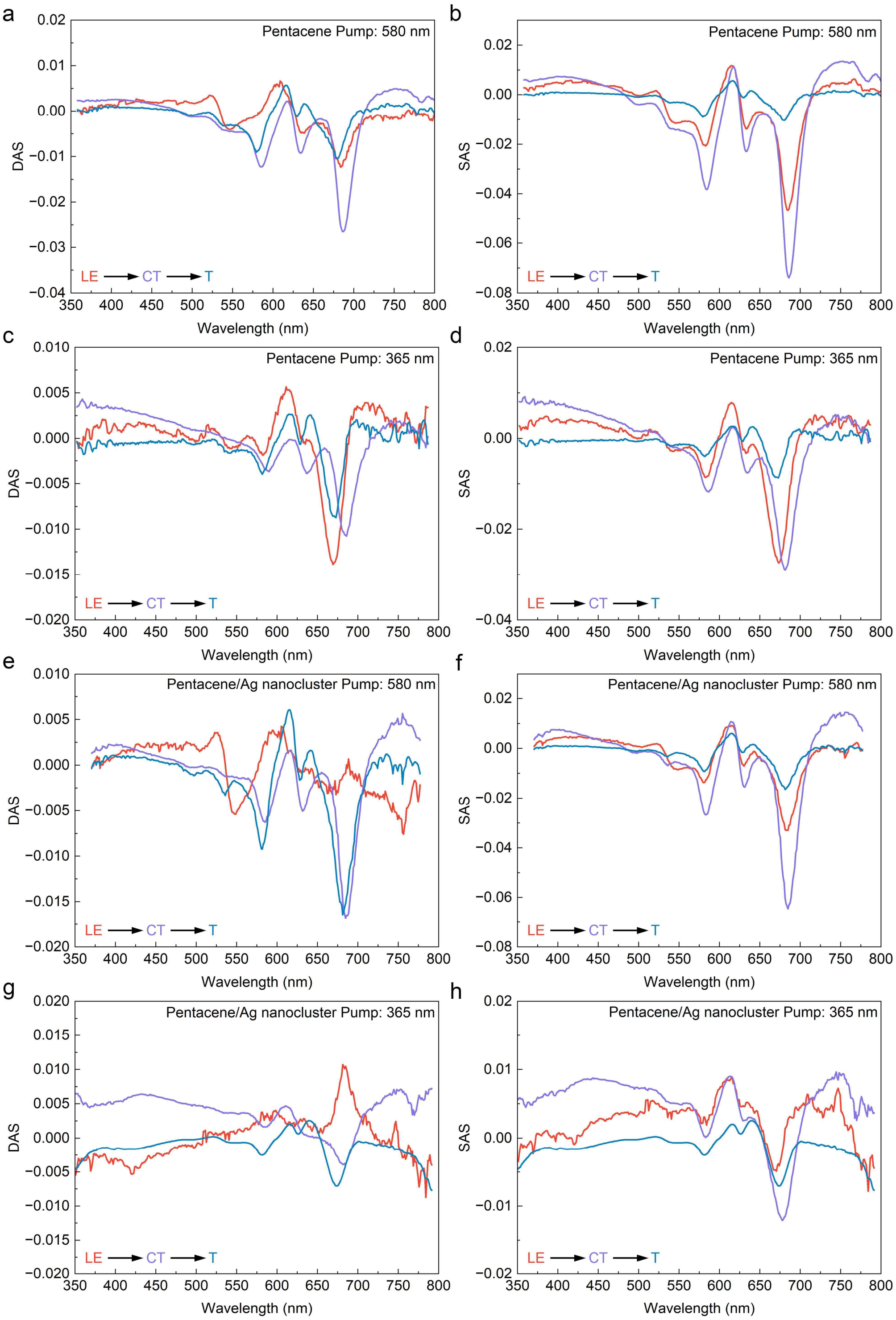

Supplementary Fig. 25 | Decay-associated spectra (DAS) and species associated spectra (SAS) analyzed from global analysis. (a, b) Pentacene film is pumped by 580 nm light. **(c, d)** Pentacene film is pumped by 365 nm light. **(e, f)** Pentacene/Ag nanocluster film is pumped by 580 nm light. **(g, h)** Pentacene/Ag nanocluster film is pumped by 365 nm light.

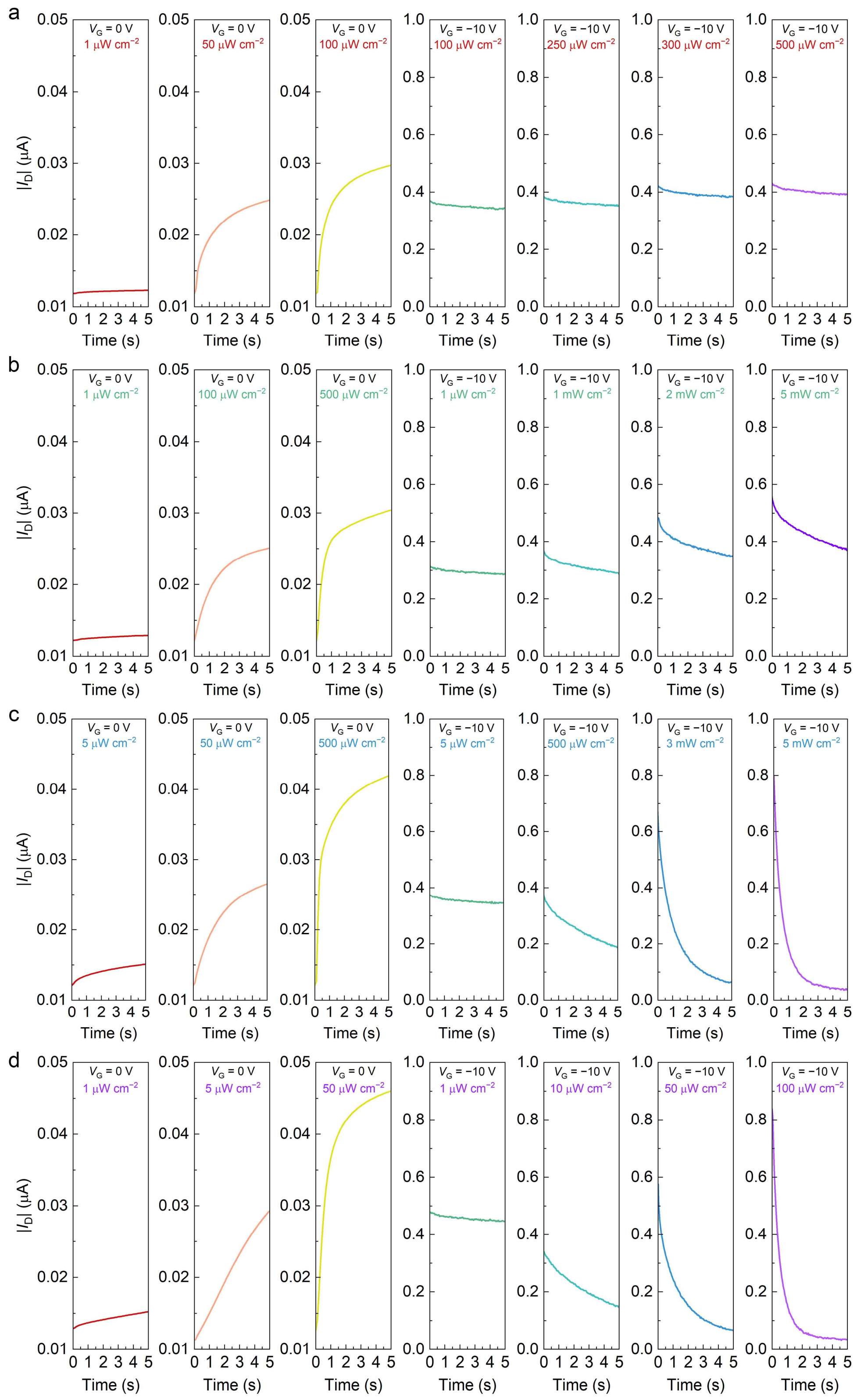

Supplementary Fig. 26 | Spectral-dependent photopic adaptation and scotopic adaptation of ACP. (a) Red (620 nm). **(b)** Green (525 nm). **(c)** Blue (460 nm). (**d)** UV (365 nm).

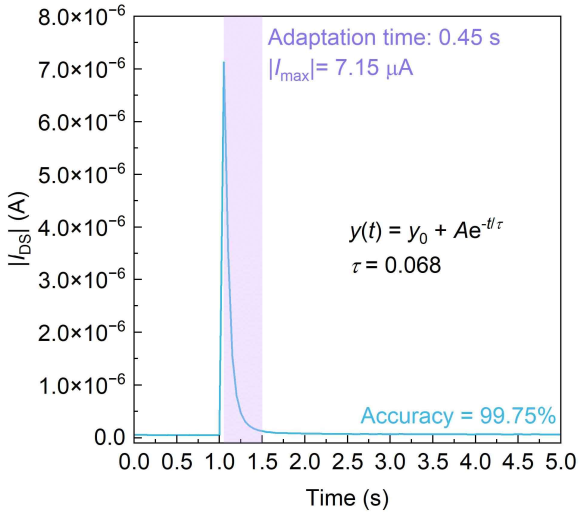

Supplementary Fig. 27 | Fast adaptation behavior of an ACP. Light (365 nm, 0.8 mW cm^{-2}) is applied for 1s, while V_G is kept constant at −30 V. According to the exponential decay equation (2), time constant (τ) for adaptation is calculated to be 0.068 s. Adaption time is also calculated to be 0.45 s.

$$y(t) = y_0 + Ae^{-t/\tau} \tag{2}$$

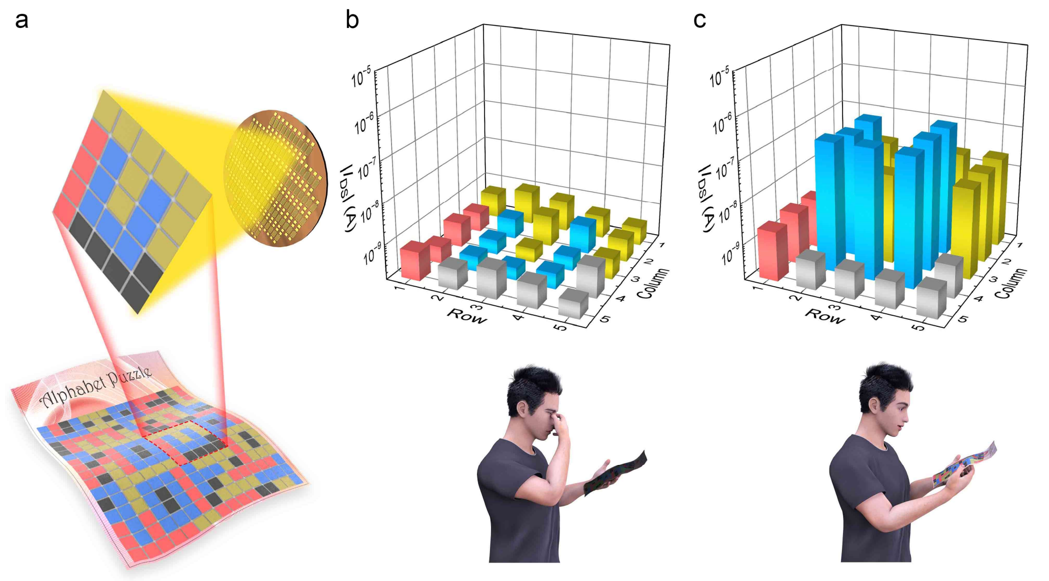

Supplementary Fig. 28 | Schematic of spectral-dependent scotopic adaptation. (a) An array comprising 5 × 5 ACP pixels perceives the pattern from a colored alphabet under incident light with various wavelengths. **(b)** Prior to illumination, current signals in 25 ACPs remain at a level of ~ 10^{-10} A. **(c)** The ACP array is exposed to 25-second-long, 2 mW cm^{-2} light pulses with various wavelengths (blue: 460 nm, green: 525 nm, red: 620 nm). Immediately after that, retention signals are extracted from the array.

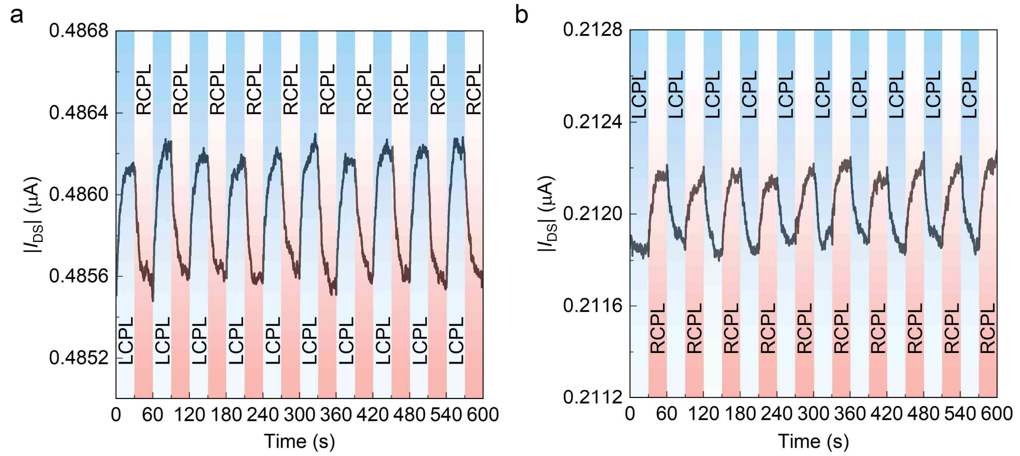

Supplementary Fig. 29 | Dynamic photoresponse of an ACP under alternatively switched RCPL and LCPL. (a) ACP with R-Ag nanoclusters. **(b)** ACP with S-Ag nanoclusters. R-Ag nanoclusters represent the chiral Ag Nanoclusters synthesized with R-type chiral ligands. S-Ag nanoclusters represent the chiral Ag nanoclusters synthesized with S-type chiral ligands. Light wavelength: 270 nm. Light intensity: 100 μW cm^{-2}.

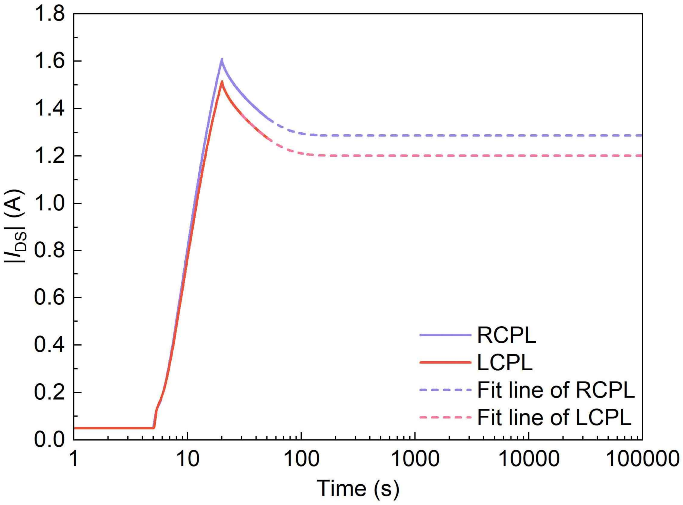

Supplementary Fig. 30 | CPL memory. After illumination of CPL (270 nm, 100 μW cm^{-2}), current decay can be well fitted by exponential decay equation (3) given as following. Hence, the current of ACP tends to be stable after 100,000 s and remains at 1.2 μA and 1.3 μA for LCPL and RCPL, respectively.

$$y = y_0 + A_1 e^{-x/t_1} \tag{3}$$

For fitted curve of RCPL (orange line), A_1=6.19975×10^{-7} ± 2.42548×10^{-9}, y_0= 1.28625×10^{-6} ± 7.16842×10^{-10}, t_1=23.77346 ± 0.14435; R-Square: 0.99992

For fitted curve of LCPL (blue line), A_1= 5.9154×10^{-7} ± 2.02882×10^{-9}, y_0= 1.20155×10^{-6} ± 6.60312×10^{-10}, t_1= 24.33669 ± 0.13736; R-Square: 0.99993

According to previous literature about CPL detectors, the distinguishing capability of circular polarized light is evaluated based on the dissymmetry factor for photocurrent, g_{ph}, which is defined by equation (4):

$$g_{ph} = (I_{LCPL} - I_{RCPL})/(I_{LCPL} + I_{RCPL}) \tag{4}$$

However, in CPL memory, I_{LCPL} and I_{RCPL} decay exponentially over time. g_{ph} increase at the beginning and finally turns constant, thereby giving a value of 0.034.

Supplementary Table 2 | Comparison of the perception range (*PR*) and adaptation time (T_{adapt}) of adaptable ACP in this work with results in literature shown in Figure 4e.

Materials	Device type	Adaptation time	Perception range (*PR*)	Wavelength
PDPP3T / PVA / PBTTT	Floating-gate phototransistor	Photopic: 1 s	40	No light
CdSe / a-IGZO / sodium (Na)-incorporated aluminum oxide	Circuit array	Photopic: 10 s<br>Scotopic: 10 s	78	465 nm, 525 nm, 620 nm
$CsPb(Br_{1-x}I_x)_3$ / MoS_2	FET based on perovskite	Photopic: 180 s	68	638 nm
Au / CsFAMA / ITO	FET based on perovskite	Photopic: 5 s<br>Scotopic: 7 s	54	white
0D-$CsPbBr_3$-QDs / 2DMoS_2	FET based on perovskite	Photopic: 3 s<br>Scotopic: 2 s	56	360 nm
PDPP3T: PCBM/PCVN/PVA/P3HT:PCBM stacking	Floating-gate phototransistor	Photopic: 2 s	120	white
$CsPbBr_xI_{3-x}$ / TIPS	FET based on perovskite	Photopic: 1200 s<br>Scotopic: 80 s	41	365 nm
$MAPbI_3$ PDs / PVPh-Li / IZO	Circuit array	Photopic: 40 s<br>Scotopic: 40 s	57	659 nm
MoS_2	2D Heterojunction FET	Photopic: 80 s<br>Scotopic: 10 s	199	660 nm
p(ACMO)-Li gated MoO_3/ Li$x$$MoO_3$	Electrochemical device	Photopic: 50 s<br>Scotopic: 50 s	16	RGB
In_2O_3 / ion-gel	Electrochemical device	Photopic: 100 s<br>Scotopic: 60 s	20	365 nm
Graphene/PbS QDs/graphene trilayer	2D Heterojunction FET	Photopic: 80 s<br>Scotopic: 10s	114	1064 nm
Pentacene / nanocluster heterostructure (This Work)	Nanocluster device	Photopic: 0.45 s<br>Scotopic: 10 s	115	UV, RGB

Supplementary Note 1: Structure and functions of mantis shrimp visual system

Part 1: Structure

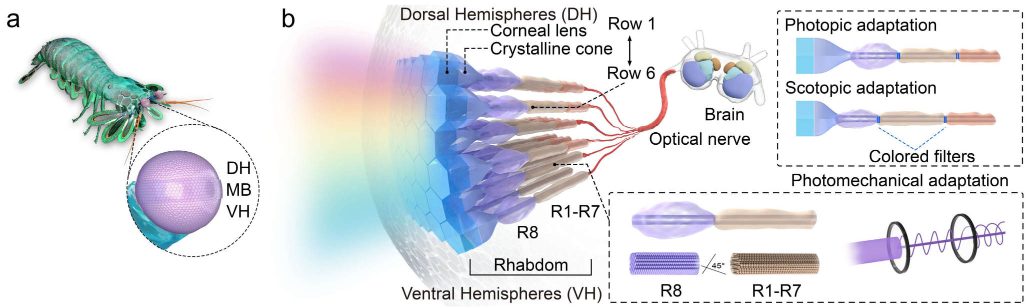

Supplementary Fig. 31 | The structure of mantis shrimp visual system. (a) A schematic of a mantis shrimp and a front view of its eyes. **(b)** Mantis shrimp visual system.

As shown in Supplementary Fig. 31, mantis shrimps possess apposition compound eyes with 16 anatomically different photoreceptor types[1]. Each eye can be sub-divided into three regions named dorsal hemispheres (DH), six-row midband (MB) and ventral hemispheres (VH), respectively[2]. MB region with six closely-spaced parallel rows of ommatidia, composed of the corneal lens, the crystalline cone and the rhabdom. The rhabdom consists of a short photoreceptor cell (R8) and seven long cells (R1 - R7), 14 types of these cells are found in MB region. Each cell has interdigitating, coplanar microvilli arranged in specific directions. In rows 2 and 3, colored filters constructed by aforementioned cells situate between rhabdom tiers. Optical nerves connect the end of rhabdom and extend to the brain.

Part 2: Functions

Color vision:

Mantis shrimps perceive the visual information through 12 channels of colors while humans only process 3 channels[2]. Each type of photoreceptor picks up a specific color, sampling a narrow set of wavelengths ranging from deep ultraviolet to far red (300 to 720 nanometers). R8 cell is sensitive to violet spectral region while R1 to R7 cells mainly response to 400 - 700 nm part of the spectrum[3].

Adaptative vision:

Mantis shrimps tunes shapes of crystalline cones and rhabdoms to execute dark/light adaptation. Cones shorten while rhabdoms extend during dark adaptation. Opposite behavior occurs in light adaptation. Photomechanical adaptation is a powerful strategy to change the account of light accepted by eyes, which plays a similar role as the

aperture of cameras or the pupil of eyes[4]. Moreover, color filters are affected by depth of water, switching light absorption range to adapt to low-light environment[1,5,6].

Circularly polarization vision:

Considering the microvillar structure of photoreceptor cells, R8 cell act as 1/4 wave plates with a fast axis parallel to their microvillar planes[7]. R8 cell enables to convert circularly polarized light into linearly polarized light, then processed by the R1-R7 cells. In rows 5 and 6, because of an angle of 45° orientation of the R8 and R1-R7 microvillar planes, the R1-R7 cells suffice to distinguish clockwise-rotating and counterclockwise-rotating electric vectors[8].

Supplementary Note 2: Nanocluster-conjugated molecule interface

Noble metal nanoclusters are stable nanostructures formed by atomically precise metal atoms protected by external ligands. Due to the quantum size effect, nanoclusters have discrete energy levels and thus exhibit excellent photophysical properties. An individual nanocluster is reported to demonstrate semiconductor characteristic behavior, but nanocluster films do not support charge carrier transport due to film inhomogeneity. Aggregation of nanoclusters hinders the effective formation of charge transport pathway within the film, which greatly limits nanocluster electronics and the study of their electrical properties. Hence, we construct a nanocluster-conjugated molecule interface (NMI) to study the optoelectronic behavior of the nanoclusters.

We selected chiral Ag nanoclusters and pentacene as the research objects of NMI. Ag nanoclusters have simple and stable structure, excellent short-wavelength absorption and extremely strong luminescence. In addition, Ag nanoclusters are enantiomorphic chiral nanoclusters that can selectively absorb circularly polarized light. These physical properties endow nanoclusters with the potential for multifunctional applications. Pentacene is featured by a simple structure, its optoelectronic properties have been widely studied. Therefore, it is very suitable to study nanocluster optoelectronic properties.

NMI is a macroscopic interface formed by the arrangement and aggregation of numerous nanoclusters and pentacene molecules. Therefore, measurement results represent the statistical behavior of electrons, holes and photo-generated excitons, which can provide guidance for nanocluster-embedded devices.

Supplementary Note 3: All-in-one system inspired by artificial nanocluster photoreceptors

Although a few mantis-shrimp-inspired artificial photoreceptors have been fabricated for polarimetric imaging and high dynamic range machine vision[9-11], none of them have noticed the significance of multi-functional integration. All-in-one system of ACP is learned from mantis shrimps that color vision, adaptative vision and circular polarization vision are integrated into a single unit (Supplementary Fig. 32). It is known to us that multifunctional visions of mantis shrimps require various photoreceptor cells, color filters and photomechanical changes of crystalline cones. Biomimetic ACP process these visions just by synergistic modulation of V_G and light on nanocluster-conjugated molecule interface as summarized in Table 1. For color vision, difference of absorption range of nanoclusters and pentacene endow ACP with distinguished response to spectral lights. For adaptative vision, nanoclusters function as light-response charge reservoir to control account of charge carriers in channel. Under the influence of nanoclusters, the channel current tends to remain in an equilibrium state. In other words, high or low charge carrier density in channel will eventually return to the intermediate value under illumination. For circular polarization vision, chirality of nanoclusters endows ACP to recognize the tiny difference of left/right circularly polarized lights. Because of charge trapping and detrapping capacity of nanoclusters, ACP even successfully achieve CPL memory and CPL adaptation.

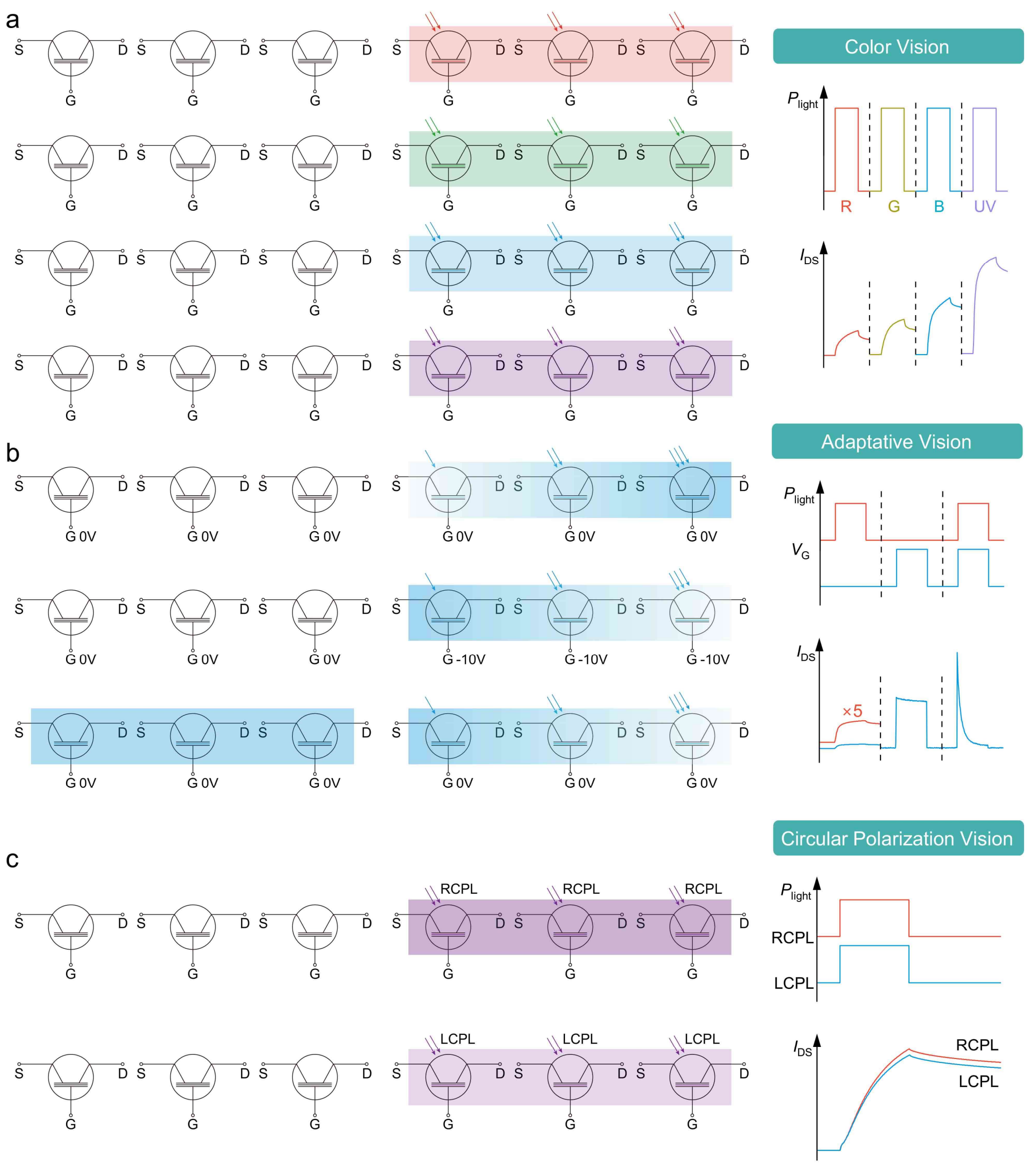

Supplementary Fig. 32 | The circuit diagram of ACP arrays. ACP arrays can imitate **(a)** color vision, **(b)** adaptative vision, and **(c)** circular polarization vision.

Supplementary Note 4: Details of nanocluster film fabrication

Here, we provide an effective strategy by using volatile solvents with high spin-coating speed to obtain high uniform and homogeneous nanocluster films. The crystallinity and solubility of nanoclusters greatly affect the uniformity and homogeneity of film formation. Nanoclusters are prone to crystallize due to their rigid core-shell structure and heavy atoms. In crystals, nanoclusters stack by non-covalent bonds, such as Van der Waals forces, hydrogen bonds, etc. to form a thermodynamically stable state. In the field of semiconductor device engineering, spin-coating is the most widespread and efficient solution processing protocol. A uniform film can be obtained by dissolving the material in a good solvent and removing the solvent through high-speed spin-coating and annealing processes. This common method promotes the development of electronics with soft materials.

We adapt the same idea and dissolve the nanoclusters in a good solvent. During the spin-coating process, the solvent is continuously removed by centrifugal force, and nanoclusters will undergo saturation and precipitation dynamically. Crystals are a common thermodynamically stable state of nanoclusters. However, in solution-processed devices, crystallized regions will produce undesirable roughness and non-homogeneity of the film surface. They also introduce defect sites that greatly affect the performance and stability of devices. Therefore, the attaining of large-area, uniform nanocluster film is the prerequisite for nanocluster electronics. Our solution is to control the kinetics of the crystallization process.

When nanoclusters are dropped onto a substrate, the substrate provides sites for heterogeneous nucleation. Crystal precipitation will undergo a competition step between nucleation and crystal nuclei growth after supersaturated state. Therefore, rapid evaporation of the solvent can inhibit the nuclei growth and afford a homogeneous film with low crystallinity. The rate of solvent evaporation depends on the solvent's boiling temperature, vapor pressure and spin-coating rate (Supplementary Table 3). Hence, it is crucial to select a volatile solvent and high spin-speed to suppress the crystallization of nanoclusters. In addition, in order to further suppress substrate-induced nucleation sites, the nanocluster solution needs to be dropped after the substrate reaches high spin-speed to reduce the contact time with the substrate.

We have searched a variety of solvents and speed conditions, and the test conditions are appended as following. The nanoclusters were all prepared at a concentration of 5 mg/ml in dichloromethane, tetrahydrofuran, and chlorobenzene, respectively. The spin-speeds were set to be 1000 rpm and 6000 rpm, respectively. The spin-time was set as 50 s to ensure that the complete evaporation of solvents. We found that nanocluster film

spun at high speed with tetrahydrofuran displays the highest uniformity. Under this condition, roughness is suppressed and controlled to be 0.45 nm. The X-Ray diffraction (XRD) result elucidates that no peaks of Ag nanocluster layer are found, validating homogeneous stacked morphology of the Ag nanocluster films (Supplementary Fig. 5). For solution in chlorobenzene, due to its high boiling point (131 °C), nanoclusters are given more growth time, leading to crystalline areas, as can be seen under optical microscope (Supplementary Fig. 6). For dichloromethane, due to its boiling point (39.6 °C) and high vapor pressure, the solvent evaporates significantly at the tip of the pipette. The solution cannot fully cover the substrate due to the rapid evaporation of dichloromethane, resulting in extremely poor film.

In summary, we found that high roughness and high crystallinity of nanoclusters greatly affect device performance. Therefore, control of nanocluster crystallization is crucial in nanocluster electronics. We provide a concise and effective strategy by using volatile solvents and high-speed spin-coating. This strategy will help researchers to reduce the time of screening conditions and promote the development of nanocluster electronics.

Supplementary Table 3 | Boiling temperature and vapor pressure of solvents.

Solvents	Boiling temperature	Vapor pressure
dichloromethane	39 °C (312.8 K)	350 mmHg
tetrahydrofuran	66 °C (339 K)	132 mmHg
chlorobenzene	131 °C (404 K)	9 mmHg

Supplementary Note 5: In-sensor light valve charge reservoir model

Nanoclusters have many discrete energy levels, and electrons fill in the lower-energy orbitals, leaving many higher-energy empty orbitals. These empty orbitals can accommodate more electrons as well as photogenerated electrons. Since the electron clouds between nanoclusters cannot effectively overlap, we believe that the occupied levels in nanoclusters are independent. According to the definition of Fermi level in semiconductors, when the electron density in nanoclusters changes, the Fermi level will change accordingly. When nanoclusters contact semiconductors to form an interface, the Fermi levels of them always remain flat. Upon applying an electric field and light illumination to this interface, the gain and loss of electrons and holes between them are conserved. Initially, the density of electrons in nanoclusters and holes in channel is low. After illumination, channel current increase indicates holes accumulation. Even after the light is removed, the current level is still higher than its initial value, which is a result of photo-generated electrons captured by nanoclusters. When electron and hole densities are prompted to a high level, channel current remains stable in the dark. Hence, nanoclusters have the ability to store and capture excess electrons. When light is shed, channel current decreases rapidly, indicating that light promotes the recombination of electrons and holes. Therefore, nanoclusters can be regarded as a reservoir, and light acts like a valve to switch the on and off of the reservoir. The in-sensor light valve charge reservoir model reveals nanocluster behaviors of charge storage, photogenerated exciton trapping and recombination.

Each material has its unique characteristics, hence the light-valve model and charge reservoir model are not universal in all kinds of nanoclusters. In fact, we propose the light-valve model and charge reservoir model to account for the photoadaptation behavior. In our work, Ag nanoclusters are revealed with adaptative response to light, charge capture, and tunable Femi energy level, hence electronic states of Ag nanocluster can be synergistically tuned by gate bias and light. These characteristics are the prerequisite for the construction of nanocluster-type photoreceptors. To address your concern, there are four requirements to achieve a successful nanocluster/organic interface. First, nanoclusters should be composed of precious metal cores that offer plenty of empty orbitals for extra electrons and function as charge reservoir. Second, aromatic ligands are required to form effective hopping pathway for charge carrier transition to π-conjugated backbones of semiconductors. Third, nanoclusters should be synthesized with high absorption coefficient. Forth, π-conjugated backbone of semiconductors should be sterically adjacent to the ligands of nanoclusters to ensure effective charge transfer. In a control experiment, we use an interlayer of tetratetracontane (TTC) to physically isolate the nanocluster and the pentacene layer, leading to suppressed photoresponse in the device. According to these rules, not every

semiconductor satisfies the requirements. Organic semiconductors with long and bulky alkyl side chains are probably not suitable for photoadaptation.

To verify our inference, we substituted pentacene by C8-BTBT as the channel semiconductor. Under a pressure of 6×10^{-4} Pa, 30-nm-thick C8-BTBT was thermally evaporated on the Ag nanocluster with a rate of 0.1 Å s^{-1}. Then, 30-nm-thick gold (Au) was thermally evaporated through a shadow mask to define source and drain electrodes. Channel width-to-length ratio was designed to be 225 (W/L = 4,500 μm/20 μm). As shown in Supplementary Fig. 33, 34, transfer characteristics curve displays a similar hysteresis under luminescence, indicating charge trapping-and-detrapping process similar to the Ag nanocluster/pentacene device. The C8-BTBT based device displays a 10^4 dynamic range of current levels. However, the overall performance of the device is inferior to that of pentacene-based device.

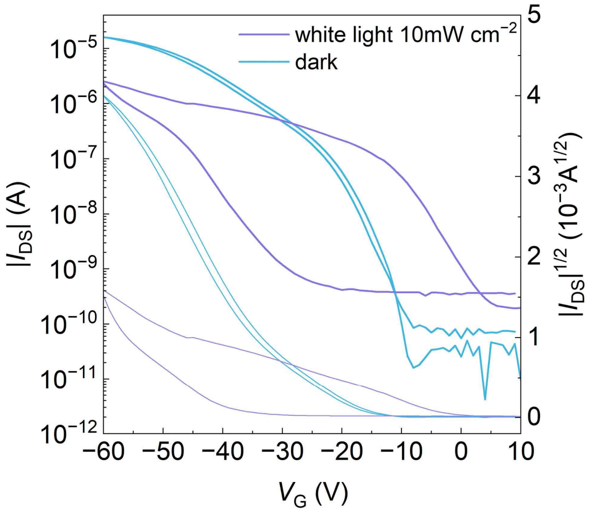

Supplementary Fig. 33 | Transfer characteristics of C8-BTBT based device. Hysteric transfer characteristics window generates under white light (10 mW cm^{-2}).

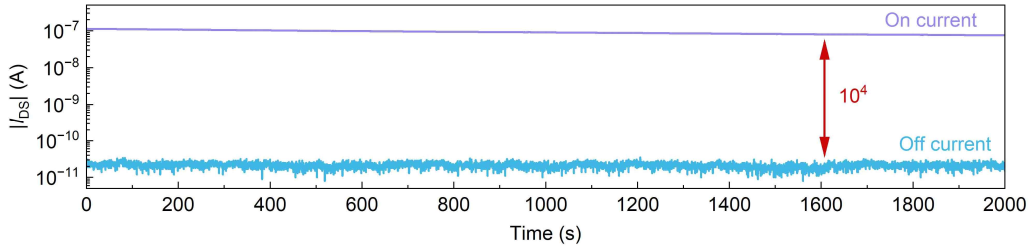

Supplementary Fig. 34 | I_{DS} Retention test of C8-BTBT-basesd device. The device can maintain the current values of two states within a timescale of 2,000 s.

Supplementary Note 6: Environmental influences on electrical performance of artificial nanocluster photoreceptor arrays

Characteristics curves of our devices have been tested under nitrogen protected circumstance at 25℃ and ambient atmosphere with various humidity at 25℃. Although the off currents of the devices have raised up in high humidity, as shown in Supplementary Fig. 35, our devices still enable to operate multifunction in kinds of circumstances. Moreover, our devices still retain good performance after being deposited in low level of oxygen and water circumstance for one year. The devices still keep retention capacity as shown in Supplementary Fig. 16.

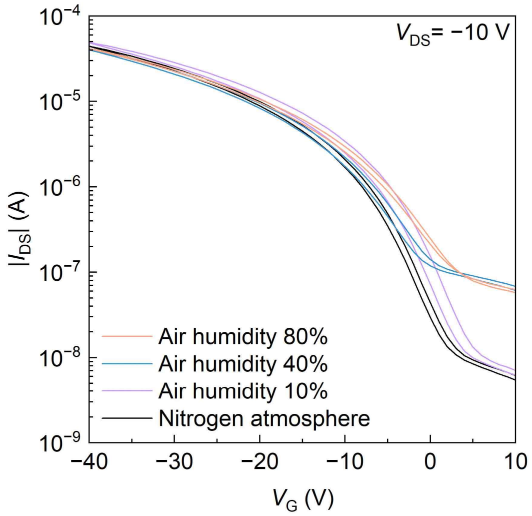

Supplementary Fig. 35 | Transfer characteristic curves of ACP devices. ACP devices can retain relatively low off currents (5×10^{-9} A) when devices are exposed to circumstance with low humidity (< 10%).

Supplementary Note 7: Indispensable characteristics of nanocluster-conjugated molecule interface

We have noticed that various nanoscale dots such as carbon dots and PbS dots have been reported with their charge-storage capacities and applications thereof in floating-gate memories and some memristors. Similar to nanoclusters, these core-shell dots mentioned above can feasibly be enabled with chirality by rational design, such as self-assembly and asymmetric ligands. Although it is possible to substitute nanoclusters by these dots as far as functionalization is concerned, nanoclusters still be indispensable theoretically and experimentally due to the following reasons.

Theoretically, the major differences between nanoclusters and nanodots are the sizes, cores and ligands. Compared with known nanodots, nanoclusters are more likely to produce thin film with low roughness due to their smaller physical size (several nm versus tens of nm), which presents an evident advantage for subsequent deposition of semiconductor films with high quality and low defects. Hence, we have selected well-dispersed nanoclusters with a diameter of nearly 1 nm and attained a large stable hysteresis under luminescence. Noble metal nanoclusters are featured by atomic centers (Au, Ag, Cu, Pt) with empty d orbitals. Such a property is anticipated to accommodate plenty of electrons under photophysical processes. Hence, cores of nanoclusters have higher charge-storage capacities than carbon dots, perovskite dots, and metal chalcogenides (MCs) dots as electron reservoir species. Another distinction is the role ligands play in nanoclusters and nanodots. Ligands are primarily adopted as colloidal stabilizers and dispersibility reagents in nanodots. In contrary, ligands directly influence energy levels of nanoclusters due their smaller physical size, which facilitates the charge transfer from protective ligands to the metal cores and thus enabling potential optoelectronic functions. In our work, we find ligands of nanoclusters function as a bridge for dissociation of electrons and holes. A ligand-assisted charge transfer process in nanocluster/organic interface has been elucidated by theoretical calculations and femtosecond transient absorption. As a brief summary, nanoclusters can hardly be substituted by carbon dots, perovskite dots, and metal chalcogenides (MCs) dots in terms of photoadaptative devices. However, we believe nanodots can be rationally designed and synthesized to achieve similar photoadaptative capabilities and they require extensive investigation from peer researchers.

To perform supplementary experiments, we have selected three common commercial quantum dots, carbon dots, PbS dots to substitute nanoclusters and tested their electric characteristics. The aqueous carbon dots were purchased from Suzhou Xingshuo Nanotech Co., Ltd and dispersed in methanol with a concentration of 10 mg mL^{-1}. The oleic acid-capped PbS QDs were purchased from Suzhou Xingshuo Nanotech Co., Ltd

and dispersed in toluene with a concentration of 25 mg mL^{-1}. Both of them were diluted to a concentration of 5 mg mL^{-1}. Parallel devices were fabricated with similar procedures in Ag nanocluster photoreceptors. As a result, these devices substituted by carbon dots and PbS dots demonstrate inferior optoelectronic performances under luminescence, as shown in Supplementary Fig. 18.

Supplementary references

1. Cronin, T. W. et al. Sensory adaptation. Tunable colour vision in a mantis shrimp. *Nature*. **411**, 547−548 (2001).

2. Marshall, J. et al. Stomatopod eye structure and function: a review. *Arthropod Struct. Dev.* **36**, 420−448 (2007)

3. Thoen, H. H. et al. A Different Form of Color Vision in Mantis Shrimp. Science. 343, 411-413 (2014).

4. Dore, B. et al. Photomechanical adaptation in the eyes of Squilla mantis (Crustacea, Stomatopoda). Italian Journal of Zoology. 72, 189-199 (2005).

5. Cronin, T. W. et al. Filtering and polychromatic vision in mantis shrimps: themes in visible and ultraviolet vision. *Phil. Trans. R. Soc. B*. **369**, 20130032 (2014).

6. Cronin, T. W. et al. Specialization of retinal function in the compound eyes of mantis shrimps. *Vision Res*. **34**, 2639−2656 (1994).

7. Gagnon, Y. L. et al. Circularly Polarized Light as a Communication Signal in Mantis Shrimps. *Curr. Biol.* **25**, 3074−3078 (2015).

8. Chiou, T. H. et al. Circular polarization vision in a stomatopod crustacean. *Curr. Biol.* **18**, 429−434 (2008).

9. Garcia, M. et al. Bioinspired polarization imager with high dynamic range. Optica. 5, 1240-1246 (2018).

10. Floreano, D. et al. Miniature curved artificial compound eyes. P Natl Acad Sci USA. 110, 9267-9272 (2013).

11. Haessig, G. et al. Bio-inspired Polarization Event Camera. arXiv:2112.01933 (2021).
